# Neocortical Connectivity during Episodic Memory Formation

**DOI:** 10.1371/journal.pbio.0040128

**Published:** 2006-04-18

**Authors:** Christopher Summerfield, Matthew Greene, Tor Wager, Tobias Egner, Joy Hirsch, Jennifer Mangels

**Affiliations:** **1**Department of Psychology, Columbia University, New York, New York, United States of America; **2**Functional MRI Research Center, Department of Radiology, Columbia University, New York, New York, United States of America; University of California IrvineUnited States of America

## Abstract

During the formation of new episodic memories, a rich array of perceptual information is bound together for long-term storage. However, the brain mechanisms by which sensory representations (such as colors, objects, or individuals) are selected for episodic encoding are currently unknown. We describe a functional magnetic resonance imaging experiment in which participants encoded the association between two classes of visual stimuli that elicit selective responses in the extrastriate visual cortex (faces and houses). Using connectivity analyses, we show that correlation in the hemodynamic signal between face- and place-sensitive voxels and the left dorsolateral prefrontal cortex is a reliable predictor of successful face–house binding. These data support the view that during episodic encoding, “top-down” control signals originating in the prefrontal cortex help determine which perceptual information is fated to be bound into the new episodic memory trace.

## Abstract 2

In humans learning to associate specific faces and houses, fMRI and connectivity analyses reveal that connectivity between extrastriate visual cortices and prefrontal cortex reliably predicts the successful encoding of visual stimuli into memories.

## Introduction

An important goal of memory research is to describe how perceptual experiences are transformed into new memories [ 1]. Human neuropsychology and neuroimaging have offered important insights into the functional neuroanatomy of episodic memory formation, revealing that it involves a network of brain regions including the medial temporal lobes (MTLs), the prefrontal cortex (PFC), and neocortical zones involved in perceptual representation of the study material or task [ 2, 3]. However, consensus has yet to emerge on how this network of regions works together to make new memories.

In particular, the contribution of the PFC to episodic memory formation has been the focus of considerable debate. Among initial proposals was the view that the PFC promotes the encoding of new associations by mediating the retrieval and elaboration of semantic information relating to the study material [ 4, 5], or that it plays a strategic role, by temporally organizing or “chunking” incoming data for more efficient storage [ 6– 8]. However, more recently, a number of researchers have argued that during episodic encoding, the function of the PFC may be to regulate input to the MTL by modulating activity in the sensory neocortex [ 1, 9– 12], which is in line with evidence from outside the domain of memory research that a key role of the PFC is to select perceptual representations on the basis of their relevance to a current goal or task [ 13, 14]. According to this view, differentiation among neural signals associated with successful and unsuccessful encoding begins as perceptual information flows through unimodal and polymodal association cortices, with a prefrontal “gain control” mechanism selecting favored perceptual codes and rejecting other information at each successive stage of the processing hierarchy [ 15]. Selected perceptual information eventually reaches MTL structures including the hippocampus, whose role is to associate (or “bind”) the details of an episode (such as objects, colors, or individuals) into a memory trace for long-term storage [ 16– 18]. “Top-down” signals from the PFC may thus play a key role in controlling which perceptual representations are fated to be included in the new episodic memory trace.

Support for this theory comes from neuroimaging studies that have identified regions of both the PFC and the sensory neocortex whose evoked hemodynamic response functions (HRFs) vary as a function of encoding success. Activation in “content-specific” sensory regions (i.e., those responsive to the material under study) has often been found to predict encoding success during the learning of new associations. For example, successful encoding of object–place associations yields activation in the lateral occipital complex (LOC) [ 19– 22], a region involved in object representation [ 23]; learning of face–name pairs leads to robust activation in fusiform gyrus regions involved in face perception [ 24, 25]; and later memory for word–color associations garners activation in visual regions close to those involved in color processing [ 26]. These studies provide circumstantial evidence for “top-down” selection of perceptual information during encoding by showing that differentiation among neural signals accompanying successful and unsuccessful associative encoding may have already begun en route to the MTL.

However, more substantial evidence in support of this view would come from the demonstration that *connectivity* between frontal and posterior brain regions has predictive power for encoding success. Preliminary evidence for fronto-posterior interactions during encoding has been offered by scalp electroencephalographic studies, in which phase-locking of neuronal oscillations in the theta-band (4–8 Hz) between the anterior and posterior neocortices has been shown to vary with encoding success [ 27, 28]. However, electroencephalography lacks the spatial resolution to determine whether connectivity is specific to the task-relevant representations, or is occurring in a widespread fashion between the frontal and occipital lobes.

In this report, thus, we turned to functional magnetic resonance imaging (fMRI), which can identify, with a high degree of replicability, discrete regions of extrastriate visual cortex that respond selectively to two classes of stimuli: faces (the “fusiform face area,” or FFA) [ 29] and houses (the “parahippocampal place area,” or PPA) [ 30, 31]. Participants viewed concurrent images of faces and houses, and were asked to intentionally encode the association between the two images (“try to remember that he lives there”). We made 2 predictions: (1) that evoked responses in the FFA and PPA would vary with encoding success; and (2) that connectivity between the PFC and FFA/PPA would also be a robust predictor of later memory for the face–house pairings. Additional connectivity analyses were used to explore the extent to which memory-related neocortical regions interacted with MTL structures presumably involved in binding together selected perceptual information.

Various techniques have been proposed to meet the challenges posed by the assessment of functional connectivity in fMRI studies, among the more prominent of which are confirmatory methods used to assess direction-specific coupling (“effective connectivity”) between pre-established regions of interest (ROIs) such as structural equation modeling [ 32] or dynamic causal modeling [ 33]. However, in the absence of a precise hypothesis as to which portion of the PFC would exhibit encoding-sensitive functional connectivity with the FFA and PPA, we opted for a more exploratory approach, in which functional connectivity was calculated between face- and place- sensitive “seed” regions and a mask comprising the entire frontal lobe. This approach is similar to that applied in a recent study exploring connectivity between the neocortex and hippocampus during episodic encoding [ 34]. The use of a mixed block/event-related design with a short study-test cycle allowed us to assess how both univariate, “event-related” hemodynamic responses varied as a function of trial type, and how multivariate, “state-related” patterns of connectivity correlated with memory performance on a block-by-block basis, a design which also draws upon recent work in memory research [ 35].

Exploring study-phase hemodynamic responses across the visual processing hierarchy, we observed that signals associated with subsequent remembering and subsequent forgetting undergo increasing differentiation as the visual signal feeds forward from the extrastriate cortex to the hippocampus. Moreover, connectivity analyses revealed that the extent to which the FFA and PPA coupled their responses with the left dorsolateral PFC was a robust predictor of encoding success. One interpretation of these data is that “top-down” signals from the PFC modulate the perceptual signal as it flows through the association neocortex, perhaps helping to select perceptual experience for conversion into episodic memory.

## Results

### Behavioral Results

Participants ( *n* = 16) intentionally encoded 20 blocks of seven face–house pairings (old pairs; see Figure 1A and 1B) and, following each block, were asked to discriminate these pairings from another seven recombined pairs drawn from the same stimulus pool (new pairs) using a five-point scale. The use of rearranged pairings as distractor stimuli at retrieval ensured that later correct responses could not be made on the basis of mere familiarity with the face or house item, but rather required the successful encoding of the face–house association. Proportions of responses to old pairs (dark gray bars) and new pairs (light gray bars) in each of the five response categories are shown in Figure 1C. Participants made high-confidence “old” responses (key 1) to old pairs on ˜51% of trials, and rarely made high-confidence false alarms (˜5% of trials).

**Figure 1:**
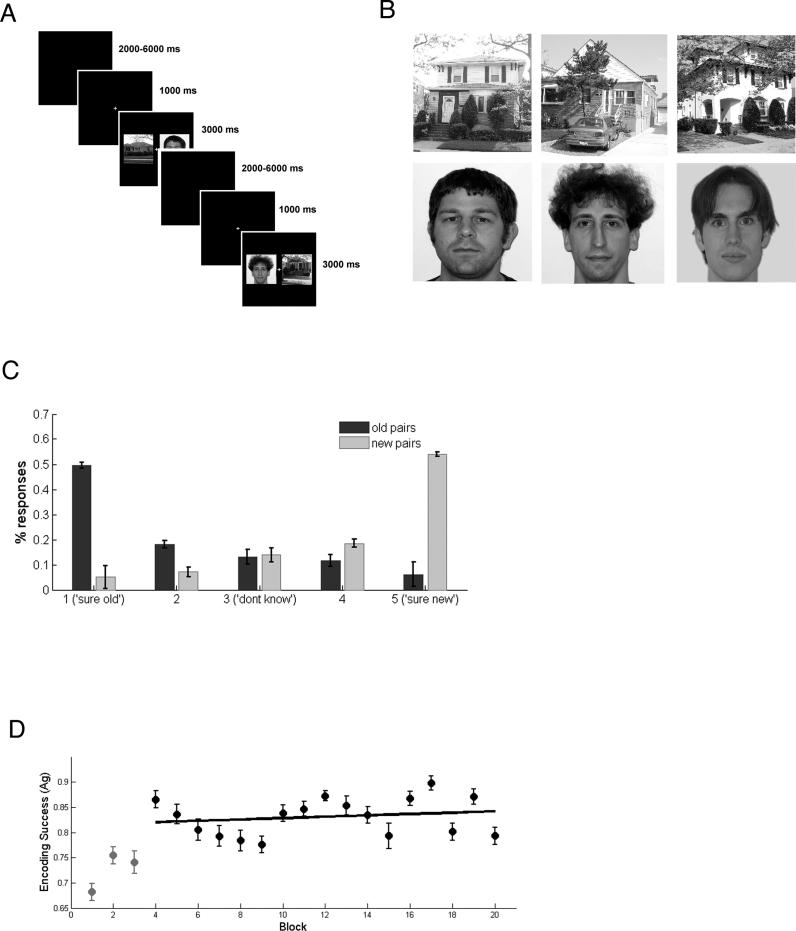
Task and Behavioral Data (A) Selected events (2 trials) comprising part of the encoding phase. At the side of each frame, the amount of time for which it was presented is indicated. (B) Examples of the face and house stimuli employed. (C) Proportion of responses to old pairs (dark gray bars) and new pairs (light gray bars) falling in each of the five reponse categories (shown on the *x*-axis: 1 [“sure old”] through 5 [“sure new”]). (D) Encoding success (the area under the ROC curve, or A _g_) averaged across participants for each block. Bars are standard errors. The first three blocks were outliers (in gray). The black line is a fitted linear trend, which was nonsignificant (F < 1).

The area under the receiver operating characteristics (ROC) curve (A _g_) [ 36] was calculated for each participant/block (range, 0.35–1.0; mean, 0.82 ± 0.15) to give an unbiased estimate of memory performance, similar to that obtained by calculating *d′* across a Likert confidence scale [ 37]. Visual inspection suggested that memory performance was reliably poorer on the first three blocks of the experiment, and this was confirmed by statistical analyses (F = 5.72, *p* < 0.01). These blocks were excluded from further analyses ( Figure 1D) after which no improvement or deterioration in performance across the 17 remaining encoding blocks was observed (F < 1).

### Event-Related Analyses in the Sensory Neocortex and MTL

In order to identify brain regions whose encoding-related responses varied with subsequent associative memory for the face–house pairs, encoding trials were sorted according to later memory performance. A regressor encoding study-phase trials for which a high-confidence “old” response was later made (key 1; later hits; 50.5% ± 17.4% of trials) was contrasted with a regressor that modeled study-phase trials for which participants later made any other response (keys 2–5; later misses; 49.5% ± 17.4% of trials). Voxels sensitive to this “difference of memory” (DM) analysis can be seen in Figure 2A, rendered onto a single axial slice. Hemodynamic responses associated with later hits (blue lines), later misses (green lines), and as a point of reference, unsorted retrieval trials (red lines) are plotted for the peak voxel from each cluster. Talairach coordinates for this voxel are given as subheadings.

**Figure 2:**
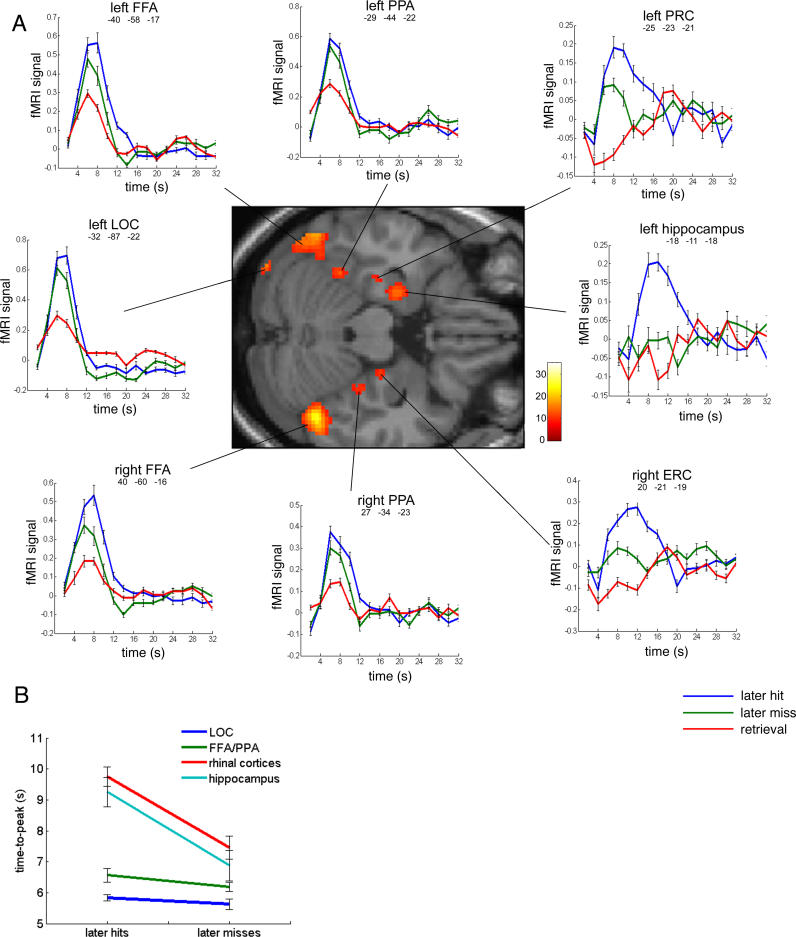
Event-Related fMRI Analyses: Sensory Neocortex and MTL (A) DM effects (voxels for which the response for successfully encoded pairs was greater than that for unsuccessfully encoded pairs) suriving a statistical threshold of *p* < 0.001 (uncorrected) are rendered onto a single axial slice of the MNI brain (a single slice was chosen for ease of visualization; voxels shown are not maxima). The scale refers to F values. Each cluster is linked with a black line to a plot of the HRFs for later hits (blue), later misses (green), and unsorted retrieval trials (red). HRFs are averaged across participants; bars are standard errors. Lineplots are titled with the name of the relevant brain region. ERC, entorhinal cortex; PRC, perirhinal cortex. Subtitles indicate the Talairach coordinates of the peak voxel for each significant cluster. (B) Time-to-peak estimates of the HRF for LOC (blue), FFA/PPA (green), rhinal cortices (red), and hippocampal (cyan) ROIs in each subsequent memory condition. Time-to-peak on the *y*-axis is in seconds.

Following correction for false discovery rate [ 38], statistically significant responses were observed in regions of the ventral inferotemporal cortex sensitive to images of faces (right FFA: F = 35.11, *p* < 0.001; left FFA: F = 14.73, *p* < 0.016) and natural scenes (right PPA: F = 11.33, *p* < 0.037; left PPA: F = 12.97, *p* < 0.024). Bilateral activation in early visual regions close to the LOC was also observed to vary with later memory (left LOC: F = 16.12, *p* < 0.012; right LOC [not shown; Talairach coordinates 43, −77, −13]: F = 17.39, *p* < 0.008). Additional DM effects were observed in the MTL, notably in the left hippocampus (F = 14.97, *p* < 0.015), the left perirhinal cortex (F = 9.72, *p* < 0.055), and the right entorhinal cortex (F = 10.5, *p* < 0.045).

### Time-To-Peak of the HRFs

Visual inspection suggested that HRFs associated with successful and unsuccessful encoding differed not only in height but also in latency, with the peak of the HRF occurring progressively later at each subsequent stage of the processing hierarchy: mean HRFs in the LOC peaked at ˜6 s, in face-sensitive regions of the extrastriate visual cortex at ˜8 s, and in the MTL at ˜10 s. Guided by a previous literature [ 39], we classed regions according to their stage in the processing hierarchy (LOC, FFA/PPA, rhinal cortices, and hippocampus), and carried out statistical analysis of the time-to-peak (the latency of the maximal hemodynamic response after stimulus) for later hits and later misses at each region. No differences in time-to-peak were observed for miss trials (all HRFs ˜6–8 s), but where an item was successfully encoded, the peak of the HRF fell at ˜9–10 s after stimulus in the rhinal cortices and hippocampus, ˜3,000 ms later than in early visual regions (˜6–7 s). Statistical analyses confirmed a main effect of region (F = 8.53, *p* < 0.001), condition (F = 9.05, *p* < 0.01), and a region × condition interaction (F = 4.0, *p* < 0.02) for HRF time-to-peak. The time-to-peak for each region and condition is shown in Figure 2B.

### Event-Related Responses in the PFC

Within the PFC, we expected to observe subsequent memory effects in the left inferior frontal gyrus, consistent with a wealth of evidence that this region is involved in episodic encoding [ 2] and in particular with the formation of new associations [ 24, 40, 41]. Indeed, DM effects were observed on the left inferior frontal gyrus in Brodmann's area (BA) 47 (F = 7.96, *p* < 0.002), peaking rather late (˜10 s after stimulus). Subsequent memory effects were also observed on the right middle frontal gyrus in BA46 (F = 12.50, *p* < 0.001). These two frontal regions, and their accompanying hemodynamic responses, are shown in Figure 3.

**Figure 3:**
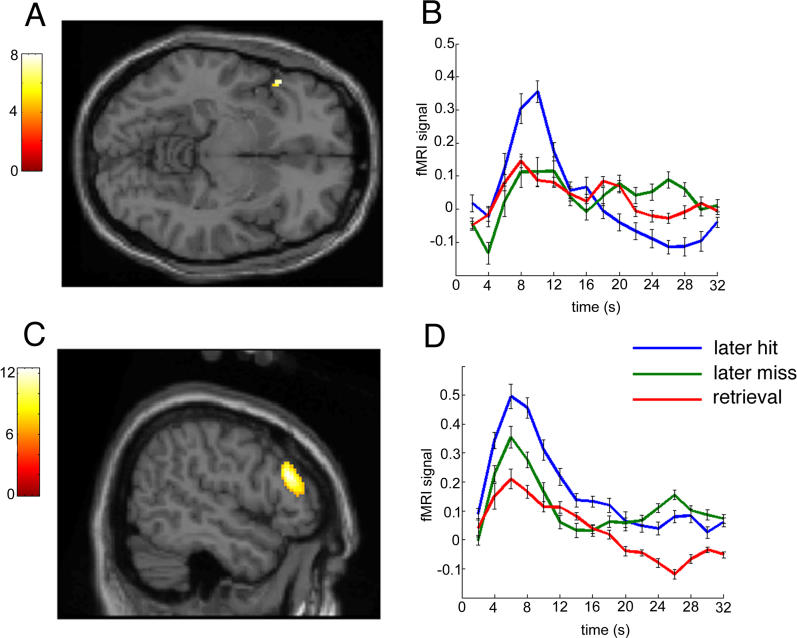
Event-Related fMRI Analyses: PFC (A) Left ventrolateral prefrontal region exhibiting DM effects (Talairach coordinates: −48, 21, −9). The scale refers to F values. (B) Mean HRFs (averaged across participants, with standard error bars) observed at this region for later hits (blue), later misses (green), and unsorted retrieval trials (red). HRF for later hits peaked at ˜10 s. (C) Right dorsolateral prefrontal region exhibiting DM effects (Talairach coordinates: 53, 30, 21). (D) Mean HRFs for this region.

### Connectivity Analyses

Our connectivity analyses were based upon the assumption that the fMRI timeseries acquired at a given voxel is composed of both “event-related” and “state-related” hemodynamic activity [ 35]. Event-related hemodynamic responses are those directly evoked by external stimulation, reflected in the characteristic positive- or negative-going deflections from baseline that typically follow stimulus onset (i.e., the HRF). State-related responses constitute part of the “residual” timeseries (which remains after the event-related responses have been removed) and reflect endogenous variation in the hemodynamic signal such as minor fluctuations in the fMRI signal about the event-related HRFs, neural activity preceding stimulus onset, or other effects which are not directly evoked by a stimulus, but which nevertheless reflect neural operations performed in the service of optimal task performance.

In order to explore the relationship between connectivity and encoding success, we calculated how dependencies among the state-related signal observed at nonadjacent voxels varied with subsequent memory performance. Our decision to explore state-related rather than event-related responses was motivated by the hypothesis that control processes contributing to memory formation may reflect a cognitive set that habituates in a stimulus-free fashion or, perhaps, interacts with stimuli to show set-specific adaptation. Both these sorts of effects are examples of set-specific endogenous variation that would not be expressed in a fixed event-related response. This view is supported by a literature suggesting that control processes are manifest in that portion of the neural signal which is not merely evoked by external stimulation: for example, control may begin in the prestimulus period [ 42, 43] or vary with the overall context of the task [ 44]. In summary, thus, the connectivity analyses described here were motivated by the hypothesis that correlation in state-related activity across the brain may be a marker for long-range neuronal cooperativity.

In order to explore how patterns of connectivity between the PFC and the FFA/PPA varied with encoding success, we extracted timeseries from the peak voxel within the FFA and PPA clusters identified by DM analyses (“seed” voxels), and from all voxels falling within a mask defining the gray matter of the frontal lobes. Following normalization and artifact removal, stimulus-evoked responses were remodelled with a finite impulse response (FIR) filter of length 32 s (bin size, 2 s), and estimates of the fit of this model subtracted from the timeseries, leaving only “residual” hemodynamic activity. Correlations between the residual timeseries acquired at the FFA/PPA, and that acquired at each PFC voxel, were calculated across the 30 datapoints constituting each encoding block. This calculation was repeated for each of the 17 encoding blocks in each of the 16 individuals scanned.

Even when event-related variance has been removed, correlation in the hemodynamic signal between brain regions can be driven by physiological artifacts such as respiration or heartbeat, making it a difficult measure to interpret independently. In order to test hypotheses of interest we thus explored how connectivity varied with encoding success (“functional” connectivity), using the area under the ROC curve (A _g_) for each block as a dependent measure. By correlating block-by-block estimates of connectivity (FFA–PFC, PPA–PFC) with encoding success (A _g_), we were able to determine how connectivity between each PFC voxel and FFA/PPA predicted later memory performance for each participant. These correlations were converted to Fisher's *z* scores, and *t* tests were conducted to determine where functional connectivity deviated from zero across the participant sample. These *t* values were rendered onto statistical maps of the PFC and thresholded in a similar fashion to event-related analyses.

### Fronto-Posterior Connectivity Results

In Figure 4A, PFC voxels exhibiting statistically significant functional connectivity with the FFA (red) and PPA (blue) are rendered onto a template brain. Adjacent regions of the left dorsolateral PFC exhibited reliable connectivity with the FFA ( *t* = 3.61, *p* < 0.003, mean *r* = 0.20) and PPA ( *t* = 3.76, *p* < 0.002, mean *r* = 0.17). The FFA cluster was centered on the left precentral and superior frontal gyri, at the junction of BA6, BA8, and BA9. The PPA cluster was located immediately inferior on the middle frontal gyrus in BA8/9.

**Figure 4:**
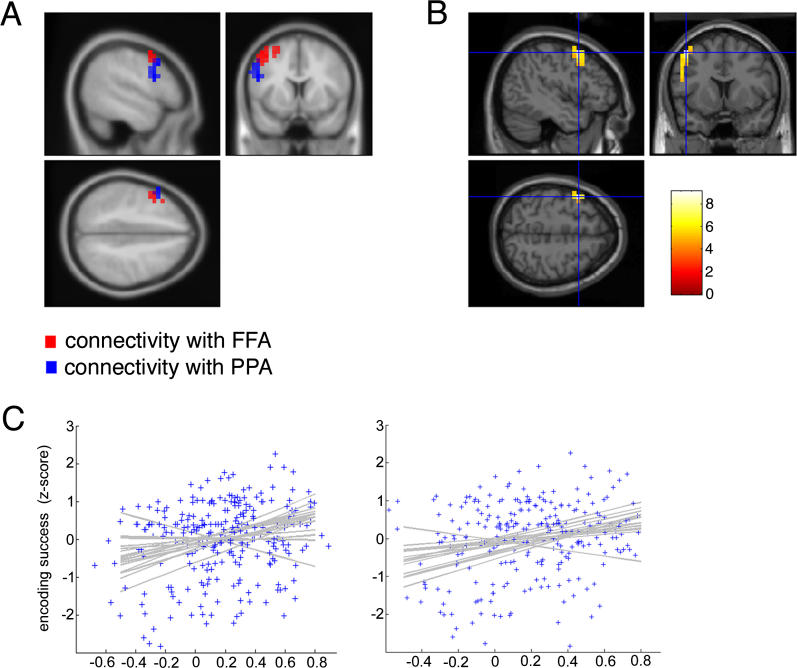
Functional Connectivity (A) Voxels exhibiting significant ( *p* < 0.01, cluster size > 62 5mm ^3^) functional connectivity with the FFA (red) and PPA (blue). (B) Voxels exhibiting significant ( *p* < 0.01, cluster size > 625 mm ^3^) functional connectivity with the average of the FFA/PPA ROI. The blue crosshairs mark the peak voxel ( *t* = 4.57, *p* < 0.001), and the color scale refers to *t* values. (C) Scatterplots of the correlation between connectivity (timeseries correlation *r* value) and encoding success (Fisher's *z* score) for the peak voxel in the PFC correlating with the FFA (left panel) and PPA (right panel). Each blue cross is one participant/block; gray lines are the fit of the correlation for each participant.

Figure 4B shows PFC voxels whose correlation with the average of the signal from the FFA and PPA was a reliable predictor of encoding success. The peak voxel within this cluster was located at Talairach coordinates −40, 6, 48 (marked with blue crosshairs in Figure 4B, falling on the left middle frontal gyrus) and was statistically significant at a higher threshold ( *t* = 4.57, *p* < 0.0004, mean *r* = 0.18). The volume of the cluster exceeding a statistical threshold of *p* < 0.001 was ˜375 mm ^3^. Figure 4C shows scatterplots of image intensity for the peak voxel in the FFA/PFC (left) and PPA/PFC (right), with best-fit lines for individual participants shown in gray. In each case, positive correlations between connectivity and memory performance can be seen in 15 of the 16 participants.

In order to demonstrate that connectivity with this region of the PFC was specific to the FFA and PPA, we performed control analyses exploring how the peak voxel in the frontal cluster (−40, 6, 48) correlated with the other regions of the processing hierarchy as defined above (LOC, rhinal cortices, and hippocampus). Connectivity between the PFC and the FFA/PPA was reliably greater than connectivity between the PFC and LOC ( *t* = 2.21, *p* < 0.05), the PFC and rhinal cortices ( *t* = 2.57, *p* < 0.03) and the PFC and hippocampus ( *t* = 2.46, *p* < 0.03), suggesting that this region of the PFC exhibited functional connectivity specifically with the FFA and PPA, and rather than more generally with the posterior brain.

### Connectivity with the MTL

Further analyses were conducted to explore patterns of connectivity linking the sensory neocortex with the MTL (including the rhinal cortices and hippocampus). The results of these analyses were, however, somewhat inconclusive. Although connectivity between the FFA and MTL was a robust predictor of encoding success ( *t* = 2.52, *p* < 0.03, mean *r* = 0.17), no reliable functional connectivity was observed between the PPA and MTL ( *p* > 0.5).

## Discussion

During performance of a task in which participants learned paired face–house associations, encoding success was found to vary significantly with activation in a network of regions comprising the PFC, the sensory neocortex, and the medial temporal lobe. In the occipital and temporal lobes, discrete waystations in the visual processing hierarchy were statistically more active for successfully compared to unsuccessfully encoded face–house pairs, including the LOC, extrastriate visual regions responsive to the face and house stimuli under study (FFA and PPA), and MTL sites including the rhinal cortices and the left hippocampus. This pattern of data replicates a number of previous studies indicating that in addition to MTL sites, content-specific activation in the sensory neocortex predicts subsequent memory [ 19– 22, 26]. Activation in the LOC, often thought to be involved in object representation [ 23], may be due to a strategy reportedly used by some individuals where they attempted to associate the face with object features drawn from the house image (such as cars, or trees), rather than the global scene.

Interestingly, estimates of the hemodynamic response from each of these regions revealed an increasing differentiation between the HRFs for later hits and later misses, as the perceptual signal processed through successive stages in the unimodal and polymodal association cortices, suggestive of content-specific selection or “gating” of perceptual information. Moreover, the peak fMRI response to later hits was not only higher but more delayed than that to later misses, an effect which increased with subsequent stages of the processing hierarchy. This effect was quantified with statistical analyses showing that the time-to-peak of the HRF increased across regions, with HRFs to hits in the rhinal cortices and hippocampus peaking ˜3,000 ms later than those in the early visual cortex. One possibility is that the staggered hemodynamic response across visual regions may reflect the cumulative effects of sustained perceptual processing during the stimulus presentation period (which lasted 3,000 ms). In other words, during relay of information through the association cortex, only those perceptual codes which undergo tonic maintenance across the encoding event survive to be processed in the subsequent stages of the hierarchy.

The data reported here also offer insight into the source of the control signals proposed to regulate activity levels in sensory neocortex. Multivariate analyses offered evidence that long-range interactions spanning the PFC and visual regions occurred during successful encoding, with a striking pattern of functional connectivity observed between the PFC and the two visual regions sensitive to the stimuli undergoing associative encoding. The FFA and PPA exhibited functional connectivity with adjacent regions of the left dorsolateral PFC stretching across cortical territory located at the junction of BA6, BA8, and BA9. One interpretation of this finding is that control signals originating in the left dorsolateral PFC selectively target visual regions during associative encoding, controlling the probability that new information is encoded by regulating activity levels in the sensory neocortex. A plausible neuroanatomical basis for exchange of neural information between the PFC and inferotemporal cortex is offered by tracing studies, which have shown that they are linked by cortico-cortical connections [ 45], although connectivity could presumably occur via either monosynaptic or polysynaptic pathways. The left predominance of this effect supports a long tradition that encoding-related processes display a left hemisphere advantage in the PFC [ 46].

A current view proposes that a generalized function of the dorsolateral PFC is to regulate activity levels in the posterior cortex, selecting for further processing those features or representations which are most relevant to the current goal or task [ 14]. It is possible that in our study, prefrontal regions were engaging in “mnemonic selection,” the selective binding of stimulus attributes chosen for long-term storage [ 47]. This view would be consistent with neuropsychological studies showing that patients with damage to the lateral PFC demonstrate deficits of associative memory that are exacerbated under conditions of high interference [ 48], as would be expected if the PFC were involved in regulating input to episodic memory [ 49]. However, our data offer only indirect evidence that the PFC mediates selection during episodic encoding. It could be, for example, that fronto-posterior connectivity covaries with another factor which predicts memory performance—for example, successful elaboration of semantic information linking the face and house images.

The model advanced here potentially offers an explanation for a puzzle in memory research: why does the left dorsolateral portion of the PFC only rarely exhibit DM effects in fMRI experiments [ 20], when neuropsychological damage to this region is highly disruptive for memory formation [ 6, 7, 50]? Our data suggest that multivariate rather than univariate hemodynamic responses associated with this region vary with encoding success, a result that may have been overlooked by researchers using only conventional analyses of imaging data. By contrast, we did observe evoked HRFs in the left ventrolateral regions of the PFC to vary with later memory, echoing a finding that is routine in neuroimaging studies of encoding. More ventral portions of the PFC may be supporting semantic elaborative mechanisms which enrich the binding between face and house representations [ 4, 10, 12]. We additionally observed right dorsolateral PFC voxels exhibiting evoked responses that predicted encoding success (but which exhibited no frontoposterior connectivity), which may be responsible for generalized monitoring or vigilance processes that keep participants' attention oriented towards the encoding task [ 51].

In addition, this theory offers a common framework for understanding the relationship between working memory and encoding. A contemporary current in memory research has argued against the “multiple-store” view, that nonoverlapping neural assemblies subserve dissociable short- and long-term representation of perceptual information. Rather, working memory maintenance may be mediated by top-down reactivation of task-relevant perceptual codes [ 11, 52, 53] via persistent reverberation in neural circuits linking the PFC with posterior regions [ 54]. The selection mechanisms leading to episodic encoding can be seen as a special case of working memory maintenance, in which favored representations are maintained tonically active for sufficient time for relevant neural information to reach the MTL for hippocampus-dependent encoding. This notion garners further support from the finding that there is a common prefrontal substrate for working memory rehearsal and episodic encoding [ 55] with a particular focus in the dorsolateral PFC [ 20, 47].

Little is known about the relationship between the fMRI signal and the underlying signatures by which neurons share information in wide-scale brain networks. However, a clue to how functional connectivity may be occurring at the neuronal level is offered by electroencephalographic studies, which have shown that patterns of neuronal oscillation thought to be important for long-term potentiation in the hippocampal formation [ 56] are also instantiated in the neocortex during successful episodic memory formation [ 27, 57, 58]. In particular, neuronal populations in the frontal and posterior neocortex exhibit theta-band (4–8 Hz) responses with consistent phase-offset during both successful encoding [ 27, 28] and working memory maintenance [ 59], a neural signature which is also observed when local field potentials are acquired from within the human MTL during encoding [ 60]. Additionally, recent evidence suggests that theta-band activity may mediate coupling between the frontal lobes and MTL during maze learning [ 61]. Theta-band activity is thus a good candidate for the carrier signal by which perceptual codes are selected for encoding. Computational models have even suggested that the push–pull dynamics of neural oscillations may be well suited to mediating target enhancement/distractor punishment mechanisms required for efficient arbitration among competing perceptual representations [ 62].

Connectivity between the FFA and MTL was also found to predict later memory, perhaps reflecting the flow of information from the ventral stream to the MTL during encoding. However, although face-sensitive regions exhibited functional connectivity with the MTL, connectivity between the PPA and MTL did not vary reliably with memory performance. One possibility is that this reflects an asymmetry in the nature of face and place representations in the brain, with place representations serving as “context” to the more central perceptual code for an object or individual (face) present in the scene [ 63]. During associative encoding a central “item” (often an object or individual) is often associated with its extrinsic “context” (such as its spatial location) [ 64]. This asymmetry makes intuitive sense when one considers that at one given moment, many objects can be embedded in a single scene, but not vice versa. The more robust FFA–MTL connectivity may reflect this priority of face stimuli during encoding. It may also be that connectivity between ventral visual regions and the MTL is better expressed in the event-related hemodynamic signal. Indeed, a recent study which explored correlation in evoked responses found connectivity between the hippocampus and visual cortex to be a robust predictor of encoding success [ 34].

The methods used here attempt to address some of the potential pitfalls of analyzing connectivity in neuroimaging datasets. We subjected the timeseries to careful artifact removal and normalization and removed task-related variance using a unconstrained model of the evoked HRF in an attempt to remove reproducibility artifacts in timeseries connectivity estimates (this is a rather conservative measure, as it is very likely that connectivity in the event-related responses also has functional significance). This meant that although posterior ROIs were selected on the basis of their HRFs, estimates of connectivity were independent of task-evoked responses, making it unlikely that connectivity results are a simple restatement of the univariate data. Finally, and most importantly, we only report connectivity that had predictive power for behavioral performance, making it very unlikely that connectivity results reflect artifacts of physiology, perfusion, or movement. This approach is related to that offered by the “psychophysiological interaction” tool in Statistical Parametric Mapping [ 65] in that both assess how functional connectivity varies with a change in experimental context (in this case, later memory performance).

Several aspects of our data suggest that this approach was successful. Connectivity results did not simply repeat univariate data: for example, functional connectivity results in the PFC were focused on a left dorsolateral prefrontal region where evoked responses did not reach threshold for differentiating trials on the basis of encoding success. Moreover, observed patterns of inter-regional connectivity were not ubiquitous across the brain, but closely matched proposed functional connectivity within feedforward visual pathways [ 39] or dorsolateral-inferotemporal pathways [ 45]. Indeed, control analyses indicated that that fronto-posterior connectivity seemed to specifically target tissue responsive to the face and place stimuli presented in the study phase of the experiment.

In summary, these data offer new insights into the mechanisms by which perceptual details are selected for inclusion in a new episodic memory trace, and provide support for a model in which the PFC exerts top-down control over perceptual representations in the posterior brain during episodic encoding, contributing to the transformation of perceptual experience into memory.

## Materials and Methods

### Participants

Participants ( *n* = 18, 8 females, 10 males) were neurologically normal individuals ranging in age from 19–34 years. All participants gave informed consent in accordance with Columbia University Medical Center Institutional Review Board guidelines. Two participants were excluded from the analyses, one because his memory performance did not deviate from chance, and the other due to excessive movement in the scanner (leaving *n* = 16).

### Stimuli

Stimuli were 300 × 300 pixel grayscale images of male faces ( *n* = 140) and houses ( *n* = 140). Faces came from various sources including the AR database [ 66] and houses were from photographs taken by the authors in Brooklyn, New York, United States. All stimuli were normalized to a mean scalar luminance of 0.5. Examples of face and house images can be seen in Figure 1B.

### Procedure

The experiment consisted of 20 study-test blocks in which participants intentionally encoded seven consecutive face–house pairs, and, following a short pause, were tested on their memory for these pairings. In the encoding phase, each trial began with a blank interval of variable length (range, 2,000–6,000 ms), followed by a central fixation cross for 1 s. The offset of the cross heralded the presentation of the face and house images, which appeared on the right and left of center (in a randomized fashion) for 3 s. Total trial length thus ranged from 6,000 ms to 10,000 ms, and the total length of each encoding block was ˜60 s. Participants were instructed to memorize the association between the face and the house as if they were learning that “he lived there.”

During each retrieval block, participants were presented with the seven face–house pairings they had just viewed, intermixed with seven recombinations of those same stimuli (14 trials). Each face and each house was thus presented exactly twice at retrieval, once with its partner from the encoding phase (old pair), and once with a new partner (new pair). Each retrieval trial began with a crosshair of 1,000 ms, followed by presentation of the old or new pair for 3,000 ms. Participants were asked to indicate whether they thought the pair was “old” or “new” by pressing one of five buttons, where the leftmost indicated that they were highly confident that the pair was old, the rightmost indicated that they were highly confident that the pair was new, the middle button indicated that they were unsure, and buttons 2 and 4 represented low-confidence “old” and “new” responses, respectively. To remind participants of the response mappings, a scale appeared underneath the paired images marked with the words “sure old” at the leftmost point, “sure new” at the rightmost point, and “don't know” in the center.

### fMRI data acquisition

Images were acquired with a General Electric Twin-Speed 1.5 Tesla scanner (Milwaukee, Wisconsin, United States). All images were acquired parallel to the anterior commissure–posterior commissure line with a T2*-weighted echo-planar imaging sequence of 24 contiguous axial slices (repetition time = 2,000, echo time = 40, flip angle = 60, field of view = 190 × 190 mm, and array size 64 × 64) of 4.5-mm thickness and 3 × 3 mm in-plane resolution, providing whole-brain coverage. The task consisted of four runs of 345 scans each (five blocks/run). High-resolution anatomical scans were acquired with a T1*-weighted spoiled gradient-recalled acquisition in the steady state sequence (repetition time = 19, echo time = 5, flip angle = 20, field of view = 220), recording 24 slices at a slice thickness of 1.5 mm and in-plane resolution of 0.86 × 0.86 mm.

### fMRI data: preprocessing

Spatial preprocessing and conventional univariate statistical mapping were carried out with SPM2 software (Wellcome Department of Imaging Neuroscience, University College London, United Kingdom; http://www.fil.ion.ucl.ac.uk/spm/spm2.html). Functional T2* images were slice-timing corrected, spatially realigned to the first volume acquired. The first five functional scans from each task were discarded prior to the subsequent analyses. Each participant's structural T1 image was coregistered to an individual mean echo-planar image. Transformation parameters were derived from normalizing the coregistered structural image to a template brain within the stereotactic space of the Montreal Neurological Institute (MNI), and the derived parameters were then applied to normalize each participant's echo-planar imaging volumes. Normalized images were smoothed with a Gaussian kernel of 9 × 9 × 13.5 mm full width at half maximum. A 256-s temporal high-pass filter was applied in order to exclude low-frequency artifacts. Temporal correlations were estimated using restricted maximum likelihood estimates of variance components using a first-order autoregressive model. The resulting nonsphericity was used to form maximum likelihood estimates of the activations.

### Trial classification and event-related analyses

Encoding trials were backsorted on the basis of performance on the subsequent retrieval block (DM analyses), with trials which later received a high-confidence “old” response (key 1) classified as “later hits” and all other trials (keys 2–5) classified as “later misses.” Regressors were constructed which convolved encoding and retrieval events with the canonical hemodynamic response [ 67] and its temporal derivative [ 68], and canonical/derivative regressors associated with later hits and later misses were compared at the group level using analysis of variance. For display purposes, voxels that survived a threshold of *p* < 0.001 were rendered onto the MNI brain. Although our inferences about significant hemodynamic responses were based upon a parsimonious model including two-basis functions, we used a more comprehensive FIR model with 2-s time bins (16 basis functions) to plot these responses. Only voxels with HRFs that were more positive-going for later hits compared to later misses are described in univariate analyses.

Estimates of performance across each encoding block were calculated by estimating the area by which the ROC curves for hits versus misses deviated from the diagonal, using a geometric approximation procedure [ 36].

The following procedure was used to estimate the time-to-peak of the HRF. First, the height H was estimated by finding where the derivative of the HRF was zero (excluding endpoints). If a dual peak was identified, the first one was chosen. Hence, our estimate of height is H = max |h| | h′ =0, where *h* is the FIR-derived hemodynamic response, and *h′* denotes the derivative of *h.* The time-to-peak T is defined as t | h (t) = H where *t* is time.

### Connectivity analyses

Timeseries were extracted using MarsBaR ( http://marsbar.sourceforge.net) from the peak voxel within the averaged bilateral FFA and PPA regions identified by DM analyses, and from within a mask comprising the gray matter of the frontal lobe as defined by the WFU_Pickatlas [ 69]. Image resolution was downsampled by resampling PFC voxels at 5 × 5 × 5 mm ^3^ ( *n* = 2486) in order to reduce computational demands. These timeseries were segmented into 20 blocks of 60 s (repetition time = 2 s, yielding 30 volumes/block), each corresponding to a single cycle of study-phase hemodynamic activity. Each timeseries was mean-centered for each block to remove gross variation in mean fMRI signal across blocks, such that univariate block effects could not contribute to variation in connectivity. Timeseries were windsorized at three standard deviations above and below the mean to eliminate spike artifacts. Responses evoked by the task were modeled with an FIR filter of length 32 s and bin size 2 s, and estimates of the fit of this model were subtracted from the timeseries. The Pearson's correlation between the timeseries at the FFA and PPA with every voxel in the PFC mask was calculated independently for each participant for each of the 17 blocks included in the analysis.

In order to calculate how functional connectivity varied with memory performance for each participant, the Pearson's correlation between FFA–PFC and PPA–PFC connectivity values and encoding success (A _g_) was calculated across the 17 encoding blocks. These values were converted to Fisher's *z* scores, and *t* tests were used to determine whether they deviated from zero across the participant sample for each region-region pairing. T values were written to Analyze format images using code adapted from Statistical Parametric Mapping, and visualized using xjview ( http://people.hnl.bcm.tmc.edu/cuixu/xjView). Frontal voxels which were found to functionally couple with the FFA or PPA at a threshold of *p* < 0.01 (cluster threshold *n* = 5 or 625 mm ^3^) were rendered onto the MNI brain. To identify frontal voxels which exhibited functional connectivity with both the FFA and PPA, the identical analyses were conducted for the average of the FFA and PPA signal.

## Abbreviations

BA: Brodmann's area
DM: difference of memory
FFA: fusiform face area
FIR: finite impulse response
fMRI: functional magnetic resonance imaging
HRF: hemodynamic response function
LOC: lateral occipital complex
MNI: Montreal Neurological Institute
MTL: medial temporal lobe
PFC: prefrontal cortex
PPA: parahippocampal place area
ROC: receiver operating characteristics
ROI: regions of interest
